# RACIAL AND ETHNIC DIVERSITY IN FAMILY CARE INTERVENTIONS: A CALL FOR ACTION

**DOI:** 10.1093/geroni/igad104.0466

**Published:** 2023-12-21

**Authors:** Naomi Meinertz, Jeong Eun Lee, Megan Gilligan

**Affiliations:** University of Missouri, Columbia, Missouri, United States; Iowa State University, Ames, Iowa, United States; University of Missouri, Columbia, Missouri, United States

## Abstract

In the United States, women are disproportionately represented as family caregivers and racial and ethnic minority families experience the challenges of caregiving at greater rates, such as poorer health and psychological distress. Recent research results demonstrate the effectiveness of family care interventions through improvements for caregiver depression, anxiety, and caregiver knowledge; yet most of these programs were developed for and evaluated with White families. The extent to which caregiver support interventions attend to racial and ethnic diversity is inadequate. Limited understanding of intervention results by racial and ethnic subgroups may ultimately further exacerbate disparities across groups by limiting our ability to account for any differences in treatment outcomes. Advances in research on caregiver interventions warrant an examination of the degree to which gender and/or racial differences are considered in the field of caregiver intervention research. Using a combined theoretical lens of symbolic internationalism and gender role theory, we argue for the development of care programs for racially and ethnically diverse family care perspectives. Thus, we conducted a systematic review of caregiver interventions and further explored whether common evidence-based care interventions are efficacious with non-White populations. Additionally, we emphasize the importance of developing targeted intervention programs by reviewing the impact of recently develop programs for specific target populations. This work underscores the gap in current family care interventions and identifies the need for evidence-based family care programs for diverse populations. Our systematic review will further our understanding about disparities in intervention outcomes to develop culturally appropriate interventions.

